# Validation of an ovine vesicovaginal fistula model

**DOI:** 10.1007/s00192-022-05342-y

**Published:** 2022-09-19

**Authors:** Lennart P. Maljaars, Stephen T. Jeffery, Marlou Scholten, Lisa Kaestner, Khumbo Jere, Deon Bezuidenhout, Zeliha Guler, Jan-Paul W. R. Roovers

**Affiliations:** 1 Department of Obstetrics and Gynecology, Amsterdam UMC location University of Amsterdam, Meibergdreef 9, Amsterdam, The Netherlands; 2 Amsterdam Reproduction and Development research institute, Amsterdam, The Netherlands; 3 Division of Urology, Groote Schuur Hospital, University of Cape Town, Cape Town, South Africa; 4 Department of Obstetrics and Gynecology, Groote Schuur Hospital, University of Cape Town, Cape Town, South Africa; 5 Cardiovascular Research Unit, University of Cape Town, Cape Town, South Africa

**Keywords:** Biomechanics, Histology, Large animal model, Surgical technique, Transvaginal approach, Vesicovaginal fistula

## Abstract

**Introduction and hypothesis:**

A representative, large animal model of vesicovaginal fistulas is needed for the training of surgeons and for the development of new surgical techniques and materials for obstetric fistula repair.

**Methods:**

The safety, feasibility, and reproducibility of vesicovaginal fistula creation were studied in 4 adult female sheep. A 1-cm fistula was created between the vagina and the bladder through a transvaginal approach. The defect was allowed to heal for 8 weeks and the animals were then euthanized. The primary outcome was the fistula patency. Secondary outcomes were fistula size, urogenital dimensions, urodynamic evaluation, histology (inflammation, vascularization, collagen deposition) and biomechanical characteristics of the fistula edge (stress at break, maximum elongation, and stiffness).

**Results:**

The transvaginal creation of a vesicovaginal fistula was safe. All animals survived the surgical procedure and follow-up period, without complications. Three of the four animals demonstrated a patent vesicovaginal fistula after 8 weeks. Baseline data are provided of the urogenital dimensions and the urodynamic, histological, and biomechanical characteristics of the model.

**Conclusions:**

The ewe is a safe, feasible, and reproducible model for vesicovaginal fistulas. The model can help to study new techniques and materials to boost surgical innovation for vesicovaginal fistula repair.

**Supplementary information:**

The online version contains supplementary material available at 10.1007/s00192-022-05342-y.

## Introduction

Obstetric fistulas are a devastating consequence of obstructed labor and result in uncontrollable and continuous leakage of urine or feces. Prolonged compression of the visceral organs in the pelvis by the obstructed fetus results in tissue ischemia, necrosis, and fistula formation [1]. The vesicovaginal fistula is the most common type and constitutes a defect between the bladder and vagina. Approximately, 2.0–3.5 million women suffer from obstetric fistulas, mostly in Sub-Saharan Africa and Asia, where there is limited access to obstetric care [1–4]. The majority of these women require surgical repair. Unfortunately, most women with an obstetric fistula will not receive surgical repair in their lifetime [5]. Expert fistula surgeons are scarce, surgery is expensive and time consuming, and the access to surgery is very limited [3, 5–9]. Currently, there is an enormous surgical backlog owing to the imbalance between the approximately 10,000 fistula repairs performed per year and the incidence of 50,000–100,000 new cases per year [1, 2, 4, 10]. Although prevention through adequate antenatal care and the availability of obstetric emergency units is the ideal long-term strategy to eradicate obstetric fistulas, there remains a large burden of untreated obstetric fistulas [1, 8]. The scale of the surgical backlog demands surgical innovation, better training programs, and upscaling of the number of fistula repairs completed per year to relieve the suffering of patients with obstetric fistulas.

A representative, large animal model of vesicovaginal fistulas can facilitate the training of surgeons and the development of new surgical techniques and materials for vesicovaginal fistula repair.

Two previous studies have reported the validation of a large animal model for obstetric fistula [11, 12]. Both studies concluded that it was a suitable model for surgical creation and repair of vesicovaginal fistula. In a pig model, a vesicovaginal fistula was created through a trans-vesical approach and an abdominal repair was performed thereafter [11]. In the pig model, spontaneous closure of the fistulas was reported owing to rapid tissue healing and inadequate fixation of the ureteral stents that were placed in the created fistula. In a sheep model, a circumferential defect involving the urethra was created and the outcomes of urethral length and closing pressure were assessed [12]. To improve understanding of the model, which includes a defect between the vagina and the bladder, and to gather more baseline data (anatomical, urodynamic, histological, and biomechanical), we designed a large animal model for vesicovaginal fistula. The Dohne Merino ewe was selected for the model in this study because of the good comparability between the ovine and human vagina, the previous experience with ovine vaginal surgery in our research group, and the absence of spontaneous closure of the fistula in the previously reported sheep model [12–14]. Our main goal was to evaluate the safety, feasibility, and reproducibility of a large animal model for vesicovaginal fistula. In addition, we evaluated the urogenital dimension and the urodynamic, histological, and biomechanical characteristics of the created vesicovaginal fistula. In this study, we aimed to validate the model for the training of fistula surgeons and for future research on innovative techniques for surgical repair of vesicovaginal fistulas.

## Materials and methods

Four female, multiparous, Dohne Merino sheep (between 5 and 6 years old), weighing between 65 and 90 kg, were used to create a vesicovaginal fistula. This study was designed to assess the feasibility and not to demonstrate statistical differences in outcomes. Therefore, the sample size was based on convenience and no control group was used. Ethical approval was obtained from the Animal Ethics Committee of the Faculty of Health Sciences at the University of Cape Town, South Africa (#021-007). The animals were handled according to the South African National Standards for the care and use of animals for scientific purposes (SANS 10386:2008).

### Surgical procedure and welfare monitoring

All animals were sourced by and housed at an experimental farm with vast experience in the handling and care of large farm animals. All the facilities required to conduct this study, including a surgical theater, were present at the farm. Ewes were housed in an outdoor pen with straw bedding, which was replaced daily, and with free access to food and water. Three days prior to surgery, the animals were moved to an indoor pen to acclimatize. Animals were starved 18–24 h before surgery to prevent aspiration and bloating. Water was withheld for no more than 12 h. A pre-anesthetic examination was performed before surgery.

General anesthesia was induced with midazolam (0.3mg/kg), buprenorphine (0.01mg/kg), and ketamine (3mg/kg) via an intravenous catheter into the cephalic vein. After induction, the animal was moved to the surgical trolley and placed in the lithotomy position. Endotracheal intubation was performed and continuous inhalation of oxygen and isoflurane (1–2%) by spontaneous breathing was given to maintain anesthesia. Ringer lactate (5–10 ml/kg/h) was given as intravenous fluid. Emergency resuscitative drugs (lidocaine, atropine, adrenalin, and doxapram) and a stomach tube could be used at the discretion of the veterinarian. The prophylactic antibiotic regimen consisted of intramuscular ceftiofur (1 mg/kg), once peri-operatively, and once daily for 2 days post-operatively. Post-operative pain management was achieved with meloxicam (1 mg/kg) subcutaneous injection administered high on the neck behind the ear on 3 consecutive days. During surgery, the animals were monitored for depth of anesthesia and cardiovascular stability (heart and respiratory rate, body temperature, oral mucosal color, and capillary refill time). The parameters were recorded on an anesthetic monitoring sheet. A registered veterinarian managed the care of the sheep peri- and post-operatively.

**Fig. 1:**
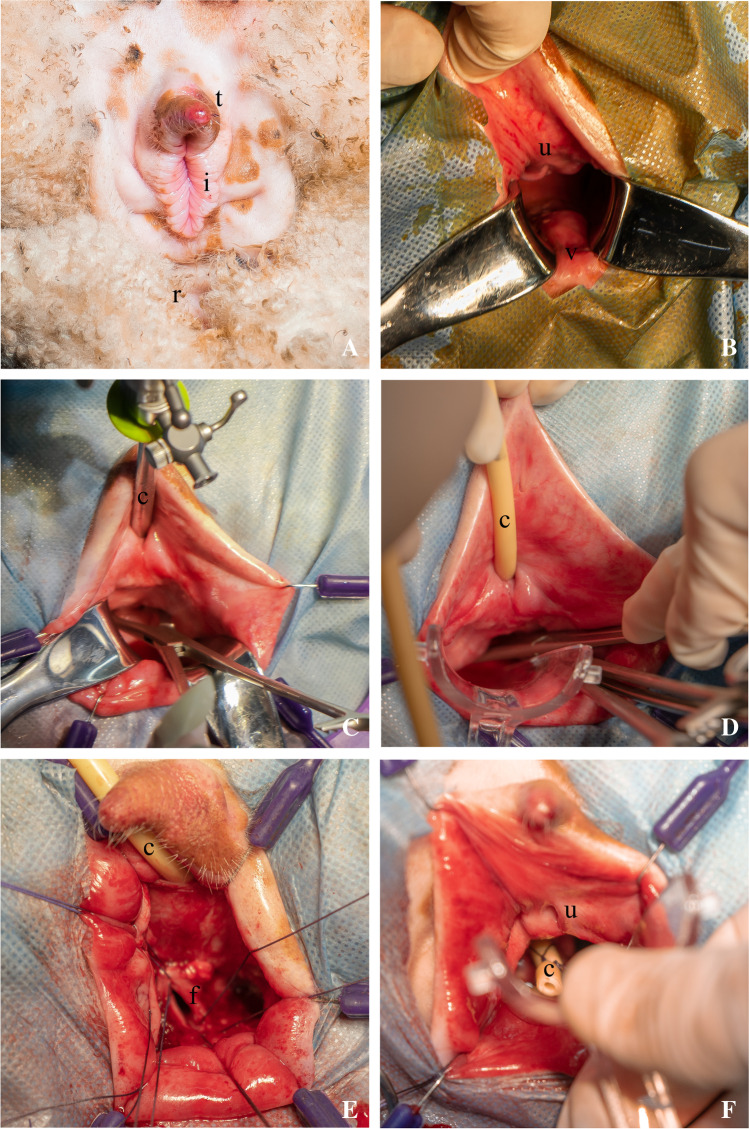
Ewe anatomy and surgical procedure. **A** External genital anatomy of a ewe with the ventral tip (*t*) of the vulva, introitus (*i*) with labia and rectum (*r*). **B** Internal genital anatomy of a ewe with the urethra (*u*) and vagina (*v*). **C** Cystoscope (*c*) and vaginal localization of the bladder. **d** Placement of a catheter (*c*) in the urethra and mid-line incision of the anterior vaginal wall. **e** Creation of a fistula (*f*) and suturing of the vaginal wall to the bladder wall, catheter (*c*) in the urethra. **f** Securement of a catheter (*c*) in the created fistula opening, above the urethra (*u*)

After induction of anesthesia, pelvic examination, urethral length measurement, and cystometry were performed (Fig. 1a, b). Standard surgical principles that are used in humans were also applied during the surgery. All procedures were executed by the same surgeon, who has extensive experience in pelvic surgery in both humans and sheep. The surgical field was disinfected with povidone-iodine 7.5% and draped according to strict surgical principles. The vaginal dimensions were measured in situ during the pre-anesthetic pelvic examination. The urethral length was measured by marking the catheter in the bladder at the external urethral opening and measuring the distance between the balloon and the marking. Cytometry was performed by connecting two intravesical catheters and one rectal catheter to a urodynamic testing device (Urocomp 2000-U, version 1.5.1, Status Medical Equipments, Satara, India). The bladder was filled at 100 ml/min with sterile saline. The infusion was stopped when there was a sharp increase in detrusor pressure (±50 cm H_2_O). The bladder was emptied, and the total volume was recorded. All measurements were repeated twice for accuracy. Cystoscopy was performed to assess the bladder anatomy and the ureteral orifices (Fig. 1c). The anterior vaginal wall was injected with a local anesthetic and vasoconstrictive agent (xylocaine with adrenaline 2% 1:200.000). To determine the correct location of the fistula the bladder was punctured transvaginally, using cystoscopy. A sharp midline incision was made and the paravesical space was dissected both sharply and bluntly toward the puncture in the bladder (Fig. 1d). The bladder was filled with sterile saline and was punctured with a sharp knife to create a defect of approximately 1 cm. The dissected edges of the bladder were sutured to the edges of the vagina to keep the fistula open. A Foley catheter (16 French) was place through the created defect and was fixated in place with sutures (Fig. 1e, f). The catheter was used to keep the fistula tract open and to induce inflammation by introduction of a foreign material. To reduce possible irritation of the catheter and prevent it from being pulled out by the animals, it was cut to approximately 5 cm in length. All animals were placed in the indoor pens for at least 24 h until full recovery from anesthesia. The animals had free access to food and water. After recovery, the animals were released into the covered outdoor pens, an area completely covered by a roof and enclosed on three sides by walls. Animal welfare, urine output, and signs of infection were monitored daily during follow-up. Weight was monitored once a week.

The created fistula was allowed to heal for 8 weeks. This period is in accordance with the primary and secondary healing stages in sheep, based on previous studies [15–17]. The catheter was removed under light sedation (midazolam (0.3 mg/kg) and buprenorphine (0.01 mg/kg)) after 4 weeks. Eight weeks post-operatively, the animals were macroscopically examined for color, swelling, and other signs of inflammation, and necrosis; and images were taken. Hereafter, the animals were euthanized by intramuscular injection of xylazine (0.1–0.2 mg/kg) and ketamine (5 mg/kg), followed by sodium pentobarbitone (20 ml; 200 mg/ml) via an intravenous catheter in the cephalic vein. Death was confirmed by rigor mortis. The bladder and vagina were resected *en bloc*.

### Outcome measurements

The primary outcome was the fistula patency after 8 weeks. The patency of the fistula tract was assessed by flushing it with sterile saline. Secondary outcomes were the fistula size, anatomical dimensions, urodynamic assessment, histology, and biomechanics. Images were taken of the fistula to record the size. The harvested vagina and bladder tissues were processed for histological and biomechanical evaluation. The bladder was dissected off the vagina and the vaginal canal was opened over the posterior wall. Vaginal samples were taken of the left and right fistula edge and at a distal control site. Histology of the left and right fistula edge and a control sample were stained with hematoxylin and eosin (H&E) to assess foreign body giant cells (FBGCs) and vascularization. Masson’s trichrome staining was used to assess collagen deposition. Five randomly chosen non-overlapping images per sample were scored at ×40 magnification. Two semi-blinded researchers scored the slides; discrepancies were resolved by consultation of a third, senior reviewer. The scores for FBGCs or vessels per high-power field were 0 = none, 1 = 1–5, 2 = 6–10, and 3= >10. Collagen deposition was scored as 0 = none, 1 = mild, 2 = moderate, and 3 = abundant [18]. Biomechanics of the left and right fistula edge and the control sample were assessed with uniaxial tensile testing using the Instron ® Merlin™ Series 5500 Testing System (Instron®, Norwood, MA, USA). Prior to testing, the frozen samples were thawed overnight and were kept in phosphate-buffered saline (PBS) at room temperature (15–25°C). The samples were clamped tension-free and tested until failure at 10 mm/min with a 2000N load cell, without preloading. Maximum stress and strain were derived from the stress–strain curves. Stiffness was calculated using the slope of the linear part of the load-displacement curves [13].

### Statistics

All data were assessed for normality. Data were reported with appropriate measures of central tendency. Groups of interest were compared using an unpaired *t* test for continuous data or Mann–Whitney *U* test for non-normally distributed data. Statistical significance was defined as a *p* value <0.05. Analysis was performed using Prism GraphPad, version 9.0 (GraphPad Software, San Diego, CA, USA) and IBM SPSS Statistics, version 26.0 (IBM, Armonk, NY, USA).

## Results

**Fig. 2:**
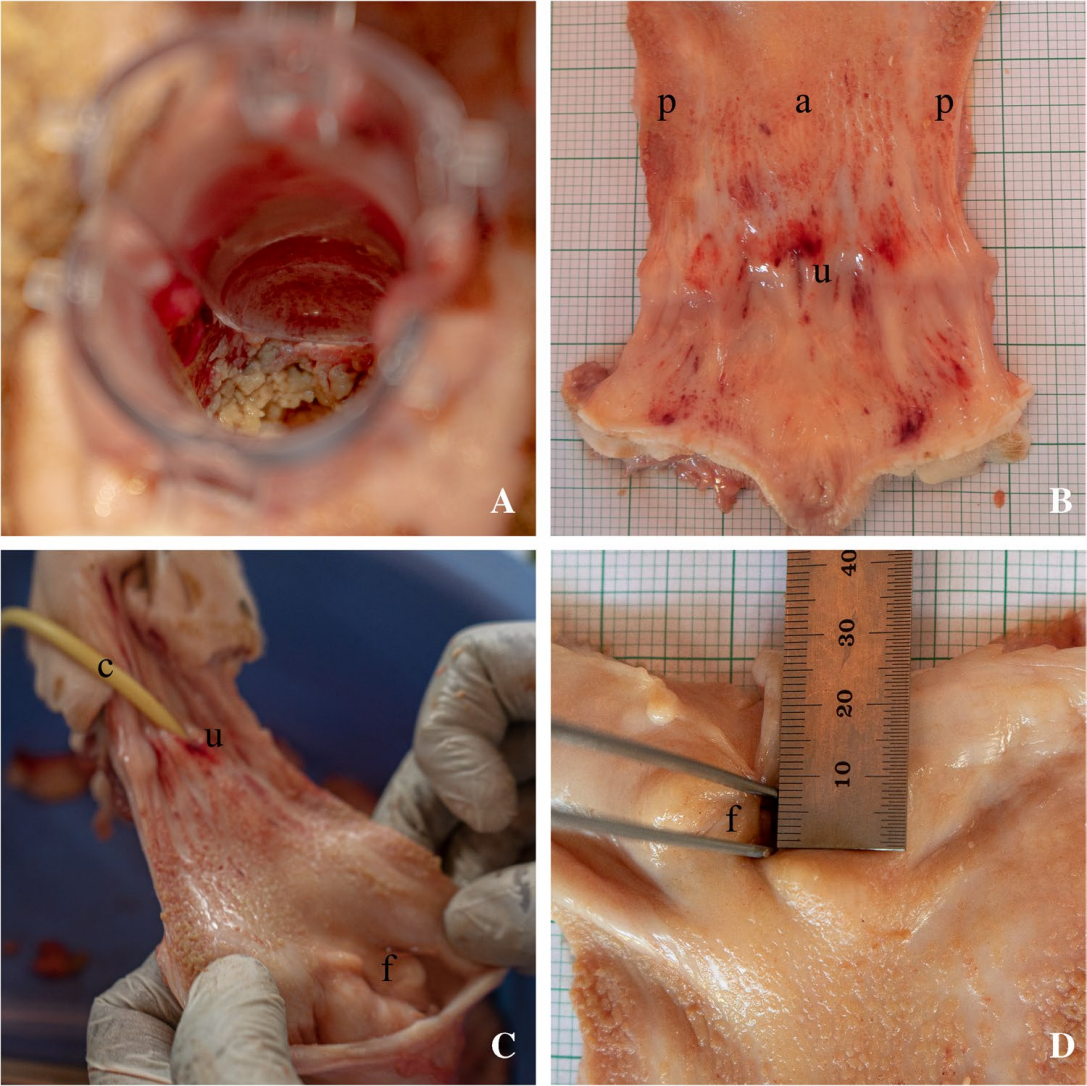
Macroscopic fistula assessment. **A** Macroscopic inspection of the vaginal canal showing necrotic discharge at the site of the fistula. **B** Mild inflammation of the distal vagina, around the urethra (*u*); the middle part of the specimen consists of the anterior vaginal wall (*a*) with both lateral edges forming the posterior vaginal wall (*p*). **C** Fistula (*f*) patency check by flushing saline through the urinary catheter (*c*) that is placed in the urethra (*u*). **D** Fistula (*f*) in the proximal part of the vagina, 8 weeks after creation of the defect

All animals survived during surgery and follow-up. No complications occurred during surgery or in the post-operative period. All sheep gained weight during follow-up, indicating their good condition. All baseline measurements and surgical characteristics are shown in Table 1. The median bladder size was 245 ml, with a range between 200 and 502 ml. The average length of the urethra and vagina were 7.3 (±1.7) and 12.8 (±2.1) cm respectively. The average duration of the surgical creation of a vesicovaginal fistula was 54 min. The surgery was challenging owing to the length of the urethra and small vaginal caliber. The vision and size of the surgical work field were limited as the access to the bladder was in the narrow, proximal part of the vagina. Cystoscopy was used to determine the correct location of the fistula between the bladder and vagina. A fistula of approximately 1 cm could be created in all animals. Blood loss was negligible and all animals were awake and standing within 1 h after the intervention. After 4 weeks, the catheter was removed from the fistula opening. In one case, the catheter was noted to have dislodged prematurely. Eight weeks after the creation of the vesicovaginal defect the animals were euthanized. Macroscopic observation of the vagina showed some necrotic discharge near the fistula due to the pooling of urine in the horizontal vagina (Fig. 2a). Mild redness, without swelling or other signs of inflammation, was seen in the vagina (Fig. 2b). Three animals had a patent fistula, in one animal the fistula tract was closed (Fig. 2c, d). The fistula patency and size are shown in Table 2.

**Table 1:** Baseline measurements and surgical procedure

Sheep characteristic (*n*=4)	Data
Preoperative weight (kg)^a^	78.9 (9.4)
Postoperative weight (kg)^a^	85.0 (7.8)
Bladder capacity (ml)^b^	245 [221–465]
Urethral length (cm)^a^	7.3 (1.7)
Vaginal length (cm)^a^	12.8 (2.1)
Vaginal width (cm)^b^	3.0 [3.0–3.0]
Surgical time (min)^a^	54 (25)

^a^Mean (standard deviation)

^b^Median [25th to 75th percentile]

**Table 2:** Fistula patency and size

Animal number	Fistula patency	Fistula size (cm)	Difference (%)
F1	Open	0.8	−20
F2	Open	1.5	+50
F3	Closed	–	−100
F4	Open	0.2	−80

**Fig. 3:**
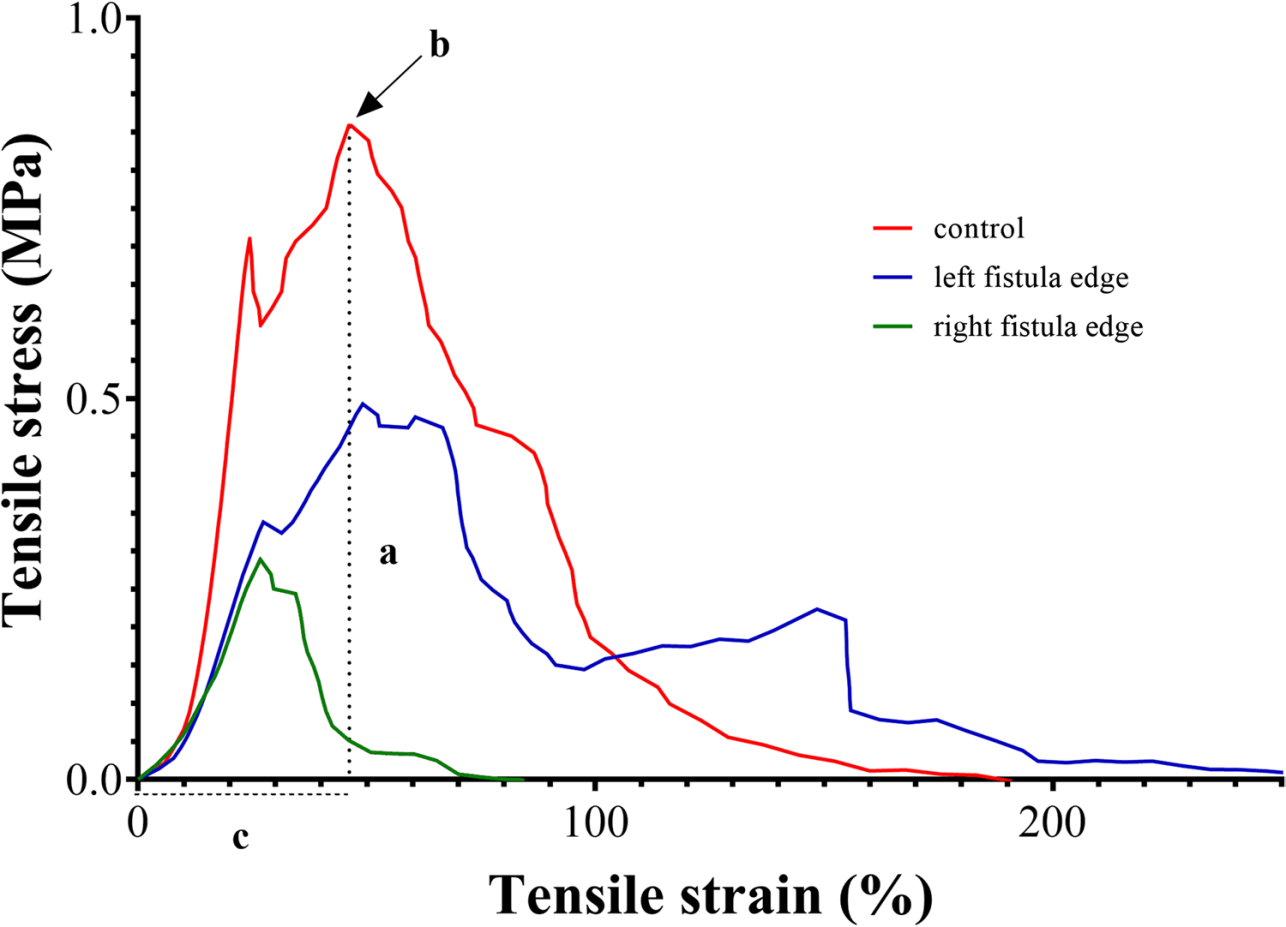
Example of stress–strain curves of biomechanical tests. Example of stress–strain curve for a control sample (*red*), left fistula edge sample (*blue*), and right fistula edge sample (*green*) of the biomechanical test of the vaginal tissue. The *interrupted line* displays the relevant biomechanical parameters of the control sample (*red*). **a** Maximum tensile stress/tensile stress at break in MPa. **b** Breaking point. **c** Maximum elongation in percentage. Note: stiffness (N/mm) is not displayed in this graph as it is the slope of the linear part of the load-displacement curve (not shown)

The outcomes of histology and biomechanics are shown in Table 3. FBGCs were near absent with no discernable differences between the fistula edges and the control sample. Vascularization scores of the right fistula edges were higher than those of the control samples, but the left fistula edges showed no significant difference from the control. Collagen deposition was higher in the left fistula samples than in the controls, but no difference was seen between the right fistula edge and control samples. Example images of the FBGCs, vascularization, and collagen deposition are shown in Supplementary Fig. 1. Stress at break was higher for the control samples than for the fistula edges, although only the right fistula edge showed a significant difference compared with controls. The maximum elongation and stiffness of the groups were comparable. Examples of stress–strain curves of the different groups, including the biomechanical parameters of interest, are shown in Fig. 3.

**Table 3:** Histology and biomechanics

	Control	Left fistula	Right fistula	p1	p2
Histology scores					
Foreign body giant cells^a^	0 [0–1]	0 [0–0]	0 [0–1]	>0.99	>0.99
Vascularization^a^	0 [0–1]	0 [0–1]	1 [1–1]	>0.99	<0.01
Collagen deposition^a^	1 [1–2]	2 [2–3]	2 [1–2]	0.02	0.20
Biomechanics					
Stress at break (MPa)^a^	0.7 [0.5–1.3]	0.5 [0.4–0.8]	0.4 [0.3–0.4]	0.20	0.02
Maximum elongation (%)^b^	59.8 (18.8)	42.5 (11.2)	45.1 (13.4)	0.33	0.14
Stiffness (N/mm)^b^	6.3 (5.2)	9.1 (2.2)	7.3 (3.7)	0.37	0.77

Foreign body giant cells and vascularization: 0 = none, 1 = 1–5, 2 = 6–10, 3 = >10

Collagen deposition: 0 = none, 1 = mild, 2 = moderate, 3 = abundant

*MPa* megaPascal, *N* Newton, *p1* comparison between control and left fistula edge, *p2* comparison between control and right fistula edge

*p* values based on unpaired *t* test or Mann–Whitney *U* test, as appropriate

^a^Median [25th to 75th percentile]

^b^Mean (standard deviation)

## Discussion

In this study, the safety, feasibility, and reproducibility of a sheep model for vesicovaginal fistulas were evaluated. The transvaginal creation of a vesicovaginal fistula was safe, with all animals surviving the surgical procedure and follow-up period. The good health of the animals was indicated by positive post-operative scores and weight gain during follow-up. No complications were seen during follow-up except for some necrotic discharge and mild vaginal redness.

It was technically feasible to create a vesicovaginal defect through a transvaginal approach in all four animals. However, it was challenging because of the length of the urethra of the animals in this study, which was much longer than the reported length in the literature [12, 14, 19–21]. The surgeon’s vision was impaired by the narrow, proximal vagina and it was therefore, more difficult to precisely determine the size of the defect. To overcome the limits of the surgical field during the creation of the defect, the light of the cystoscope was used to determine the correct location of the bladder from the vagina, after which a syringe was used to puncture the bladder transvaginally. When considering transvaginal surgical repair of the fistula in this model, it seems technically challenging owing to the limited exposure of the surgical field. The transabdominal anatomy of the sheep bladder is more globular and has a larger intraperitoneal surface than the human bladder, which is more “boat” shaped. The contact surface between the vagina and the base of the bladder in sheep is smaller, with the vesicouterine peritoneal reflection continuing much lower in sheep. Furthermore, the urethra, which is positioned against the anterior wall, is longer because of the wide pubic symphysis in sheep. These factors have likely made it more challenging to create the vesicovaginal fistula in this model. Future strategies to improve the surgical exposure and precision of determining the defect size would be to use sheep with a lower body weight. Studies show that higher body weight is associated with larger urogenital dimensions, although age and hormonal status also influence the dimensions [21]. Furthermore, it was shown that weight differs among different breeds of sheep [19]. It is therefore important to consider these factors in this model. Creating the defect in the distal vagina, like the circumferential defect of the urethra created by Crawford et al. [12] could also be an alternative strategy to improve the surgical exposure. However, this depends on what type of defect is modeled. Last, an abdominal or laparoscopic approach could be used to create and/or repair the defect in the proximal vagina. However, this strategy limits the comparability with human obstetric fistula repair, which most often occurs transvaginally.

The procedure was reproducible with three of the four animals demonstrating a patent vesicovaginal fistula after 8 weeks. The spontaneous closure of one fistula was most likely due to the premature loss of the catheter within 4 weeks after the surgery. This can be easily corrected with better fixation of the catheter in the fistula opening or looping the catheter through the fistula and urethra and connecting both ends. In the patent vesicovaginal fistulas, we saw epithelialization of the fistula tract, most likely caused by mild inflammation owing to the catheter material and the leakage of urine [22]. The method of creating the vesicovaginal fistula in this model does not fully resemble the pathophysiological mechanism of obstructed labor with tissue ischemia, pressure necrosis, and fistula formation. This mechanism could be mimicked by using magnets to inflict local pressure necrosis; however, this method is limited by the fact that a magnet needs to be implanted and the maximum magnetic force that can be achieved [23]. To date, no literature has been published on the use of magnets to mimic the pressure necrosis of obstructed labor. An alternative method of fistula creation that has been studied is cautery [24]. However, this brings no obvious advantages over the surgical creation of the fistula. In future studies, crush injury of the fistula edges or inflation of a balloon in the bladder during surgery can be evaluated, to see if those methods better mimic pressure injury. The current model certainly mimics iatrogenic fistulas and is currently the best option for mimicking obstetric fistulas. When considering alternative models for vesicovaginal fistula surgery, none of the models has shown easier vaginal access than the ewe [11, 12, 14]. Artificial models could be an alternative option in the future; however, current models lack tissue response, vaginal pressure differences, and wound healing, and can only mimic complications to a limited extent [14]. Therefore, the need for a large animal model remains and we argue that to date, the ewe is the best option.

As for the secondary outcomes, histology, and biomechanical characteristics, the outcomes were comparable between groups. The biomechanical data showed a trend toward higher stress at break and a longer maximum elongation in the control group, although this was not significant in most comparisons. The trend can be explained by the lower tissue strength of the damaged tissue of the fistula edges and could correspond to the proliferation phase of the healing response, where tissue is more fragile and inelastic owing to insufficient synthesis of elastin and a lack of crosslinking and maturation of collagen. Histological data showed higher collagen deposition at the fistula edges, although this difference was not significant either. However, the newly deposited collagen is not fully remodeled after 8 weeks and therefore lacks normal tissue strength. Reported biomechanical characteristics in the literature vary considerably because of the viscoelastic nature of the vaginal tissue and methodological differences in the studies. However, our results were within a comparable range [21, 25, 26]. The other histology outcomes showed low FBGC counts, which was expected, as no foreign material was implanted in the sheep [18]. This was also in accordance with the lack of macroscopic inflammation seen in the sheep. Vascularization was comparable among groups.

Our study has several strengths and limitations. First, the transvaginal approach that was used to create the vesicovaginal defect mimics the human condition well. The minimally invasive approach allowed the evaluation of techniques and materials in the limited space of the vaginal canal. It can facilitate the training of surgeons in an environment resembling human vesicovaginal fistula repair. This not only applies to vesicovaginal fistulas with an obstetric origin but also to vesicovaginal fistulas after pelvic surgery, malignancy, or irradiation. Second, the Dohne Merino sheep used in this model were resilient, docile, manageable, widely available, cost-effective (around US$ 200 per animal), and ethically acceptable species for use as a large animal model. This study offers thorough baseline data of the urogenital dimensions and urodynamic, histological, and biomechanical characteristics of the Dohne Merino ewe for future research. A limitation in this study was the unforeseen long length of the urethra, which made the fistula creation challenging and the possibility of transvaginal repair of the fistula uncertain. However, this can be prevented by considering the weight and breed of the animals in future experiments. Another limitation was the spontaneous closure of one fistula. This can also be easily corrected by better fixation to the fistula edge or looping of the catheter through the fistula and urethra. Last, the histology was limited to FBCG count, vascularization, and collagen deposition. In future studies, histology should focus on elastin content and fibrotic response.

## Conclusion

In this study, we demonstrated that a sheep model for vesicovaginal fistulas is safe, feasible, and reproducible. The procedure yielded a satisfactory open fistula rate without complications in the animals. In addition, we provided thorough baseline data on the urogenital dimensions and urodynamic, histological, and biomechanical characteristics of the Dohne Merino ewe vesicovaginal fistula model. This sheep model should be further evaluated in a surgical setting to validate it as an option for studying new techniques or materials to boost surgical innovation and to train new surgeons for upscaling repair case volumes. Ultimately, we argue that this model can be of value in research aimed at the relief of the suffering of patients with obstetric fistulas.

## Supplementary information

Supplementary Fig. 1 Histology of foreign body giant cells, vascularization, and collagen deposition. **a** Foreign body giant cells indicated by the *black arrows* (hematoxylin & eosin [H&E] staining at ×40 magnification). **b** Vascularization indicated by *asterisks* H&E staining at ×40 magnification. **c** Mild collagen deposition (Masson’s trichrome [MT] staining at ×40 magnification). **d** Abundant collagen deposition (MT staining at ×40 magnification) (DOCX 3.19 MB)
