# Supplementary material for: Validation of an ovine vesicovaginal fistula model
Source: Int Urogynecol J. 2022 Sep 19;33(11):3185–93. doi: 10.1007/s00192-022-05342-y (PMC9569284; doi:10.1007/s00192-022-05342-y)
Supplement: Supplementary file 1 — Histology of foreign body giant cells, vascularization, and collagen deposition. a Foreign body giant cells indicated by the black arrows (hematoxylin & eosin [H&E] staining at ×40 magnification). b Vascularization indicated by asterisks H&E staining at ×40 magnification. c Mild collagen deposition (Masson’s trichrome [MT] staining at ×40 magnification). d Abundant collagen deposition (MT staining at ×40 magnification) (DOCX 3.19 MB) [file 192_2022_5342_MOESM1_ESM.docx]

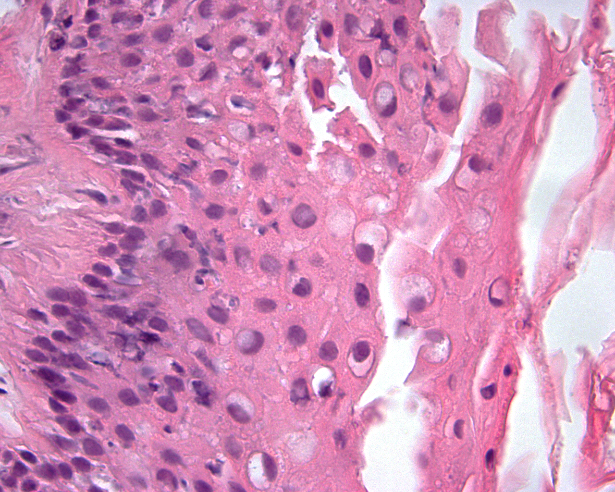

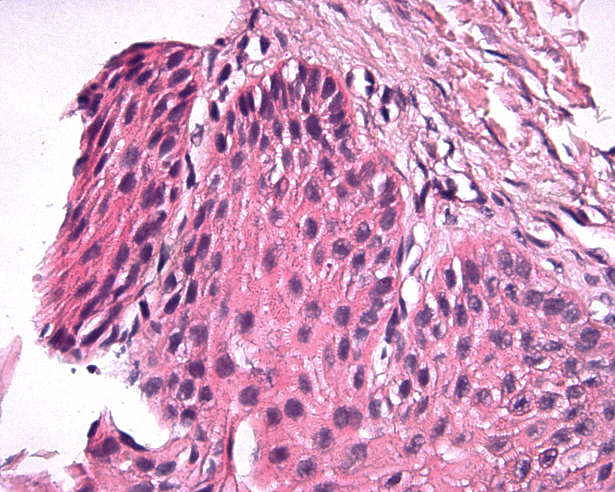


*****

*****

*****

*****

*****

**B**

**A**


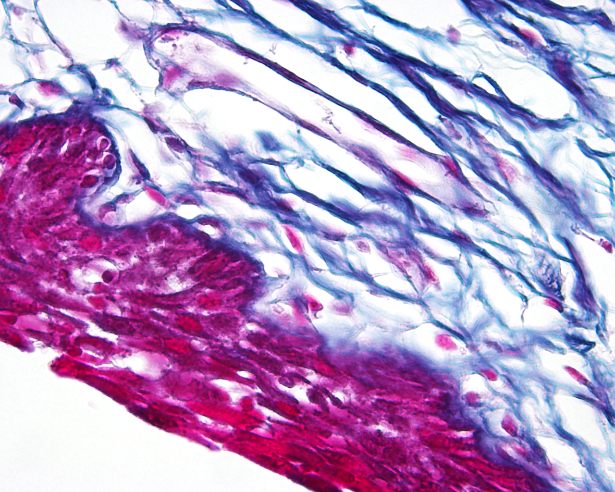

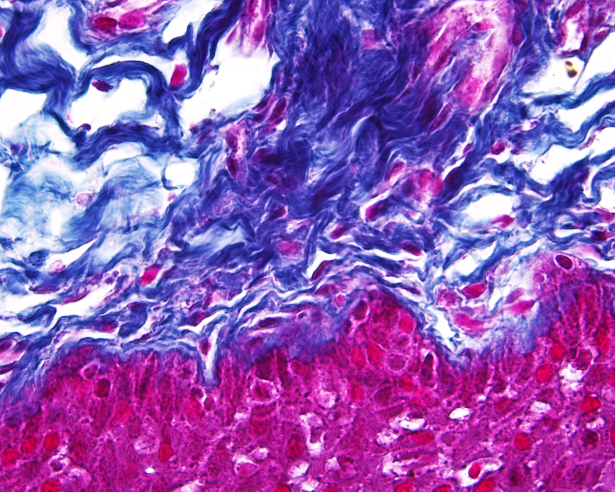


**D**

**C**

**Supplementary Fig. 1** Histology of foreign body giant cells, vascularization, and collagen deposition. **a** Foreign body giant cells indicated by the *black arrows* (hematoxylin & eosin [H&E] staining at ×40 magnification). **b** Vascularization indicated by *asterisks* H&E staining at ×40 magnification. **c** mild collagen deposition (Masson’s trichrome [MT] staining at ×40 magnification). **d** Abundant collagen deposition (MT staining at ×40 magnification).
